# The Challenge of Improving Soil Fertility in Yam Cropping Systems of West Africa

**DOI:** 10.3389/fpls.2017.01953

**Published:** 2017-11-21

**Authors:** Emmanuel Frossard, Beatrice A. Aighewi, Sévérin Aké, Dominique Barjolle, Philipp Baumann, Thomas Bernet, Daouda Dao, Lucien N. Diby, Anne Floquet, Valérie K. Hgaza, Léa J. Ilboudo, Delwende I. Kiba, Roch L. Mongbo, Hassan B. Nacro, Gian L. Nicolay, Esther Oka, Yabile F. Ouattara, Nestor Pouya, Ravinda L. Senanayake, Johan Six, Orokya I. Traoré

**Affiliations:** ^1^Group of Plant Nutrition, Institute of Agricultural Sciences, ETH Zurich, Lindau, Switzerland; ^2^Yam Improvement for Income and Food Security in West Africa Project, Research for Development, International Institute for Tropical Agriculture, Abuja, Nigeria; ^3^Laboratory of Plant Physiology, Université Felix Houphouët Boigny, Abidjan, Côte d'Ivoire; ^4^Group of Sustainable Agroecosystems, Institute of Agricultural Sciences, ETH Zurich, Zurich, Switzerland; ^5^Department of International Cooperation, Research Institute of Organic Agriculture, Frick, Switzerland; ^6^Département Recherche et Développement, Groupe de recherche sécurité alimentaire, Centre Suisse de Recherches Scientifiques, Abidjan, Côte d'Ivoire; ^7^Côte d'Ivoire Country Programme, World Agroforestry Centre, Abidjan, Côte d'Ivoire; ^8^Laboratoire d'Analyse des Dynamiques Sociales et du Développement, Université d'Abomey-Calavi, Cotonou, Benin; ^9^Laboratoire Sol Eau Plante, Institut de l'Environnement et Recherches Agricoles, Ouagadougou, Burkina Faso; ^10^Laboratoire d'étude et de recherche sur la fertilité du sol, Institut du Développement Rural, Université Nazi Boni, Bobo Dioulasso, Burkina Faso; ^11^Department of Agriculture, Field Crops Research and Development Institute, Mahailluppallama, Sri Lanka

**Keywords:** *Dioscorea* spp., soil fertility, interdisciplinarity, transdisciplinarity, innovation platforms

## Abstract

Yam (*Dioscorea* spp.) is a tuber crop grown for food security, income generation, and traditional medicine. This crop has a high cultural value for some of the groups growing it. Most of the production comes from West Africa where the increased demand has been covered by enlarging cultivated surfaces while the mean yield remained around 10 t tuber ha^−1^. In West Africa, yam is traditionally cultivated without input as the first crop after a long-term fallow as it is considered to require a high soil fertility. African soils, however, are being more and more degraded. The aims of this review were to show the importance of soil fertility for yam, discuss barriers that might limit the adoption of integrated soil fertility management (ISFM) in yam-based systems in West Africa, present the concept of innovation platforms (IPs) as a tool to foster collaboration between actors for designing innovations in yam-based systems and provide recommendations for future research. This review shows that the development of sustainable, feasible, and acceptable soil management innovations for yam requires research to be conducted in interdisciplinary teams including natural and social sciences and in a transdisciplinary manner involving relevant actors from the problem definition, to the co-design of soil management innovations, the evaluation of research results, their communication and their implementation. Finally, this research should be conducted in diverse biophysical and socio-economic settings to develop generic rules on soil/plant relationships in yam as affected by soil management and on how to adjust the innovation supply to specific contexts.

## Introduction

Yam (*Dioscorea* spp.) is a tuber crop grown by smallholders throughout the tropics (Andres et al., 2017). The most important species are *Dioscorea alata* (greater or water yam), *Dioscorea rotundata* (white guinea yam), *Dioscorea cayenensis* (yellow guinea yam), and *Dioscorea esculenta* (lesser yam) (Arnau et al., 2010). Besides being a staple consumed by 155 million people, yam is grown as a cash crop and a medicinal plant (Lebot, 2009; Sangakkara and Frossard, 2014) and has a high cultural value for some of the groups growing it (Coursey, 1981). Despite its importance, yam remains an orphan crop (Kennedy, 2003; Naylor et al., 2004).

West Africa produced 62 million tons of tuber (91% of world production) in 2014 (FAOSTAT, 2016). Until now the increased tuber demand was achieved by enlarging cultivated surfaces from 0.9 million ha in 1961 to 7.0 million ha in 2014. In the meantime mean fresh tuber yield increased only from 7.8 t ha^−1^ in 1961 to 8.8 t ha^−1^ (FAOSTAT, 2016), whereas tuber yields equal or higher than 50 and 40 t ha^−1^ were reported on research plots for *D. alata* and *D. rotundata*, respectively (Lugo et al., 1993; Asiedu et al., 1998; Chude et al., 2011; Diby et al., 2011a; Bassey and Akpan, 2015). The yam belt of West Africa spans from the humid forest where yam is cultivated for food security to the northern Guinean savanna where yam is also cultivated as a cash crop (Ndabalishye, 1995; Asiedu and Sartie, 2010). Yam is traditionally planted as the first crop, after a long-term fallow as it is considered to be demanding in terms of soil fertility (Carsky et al., 2010). In the following years, the field is cultivated with other staple crops and/or perennial crops. Yam is usually grown without any external input using own tubers as planting material (so called yam seed). In areas where land is scarce, farmers grow yam after only a year of fallow or without fallow (Maliki et al., 2012b).

Lebot (2009) reports that producers perceive soil fertility decline as a key constraint for yam production in areas under intensive use. A recent global survey classified the topic “Improving soil fertility (micronutrients, fertilizer, organic matter)” as the second most important topic to be addressed in research preceded by “Improving shelf life of yam tubers” (Abdoulaye et al., 2014). Although soil fertility degradation and inadequate plant nutrition are recognized problems (Asadu et al., 2013), little has been done to address them. In the first global conference on yam held in 2013, only seven out of a total of 115 presentations dealt with these issues (IITA, 2013).

This review discusses the importance of soil fertility for yam, barriers that might limit the adoption of integrated soil fertility management (ISFM) in yam-based systems in West Africa, and the concept of innovation platforms (IPs) as a tool for designing innovations in yam-based systems before providing recommendations for future research.

## Importance of soil fertility for yam production

We consider here soil fertility to be the result of the combination between soil properties and crop management on plant growth and tuber yield (Sebillotte, 1989; Patzel et al., 2000). The importance of soil fertility for yam was illustrated by Diby et al. (2009) who showed that tuber yields measured under the same climate, without fertilizer input, over two successive years were higher (40 t ha^−1^ for *D. alata* and 21 t ha^−1^ for *D. rotundata*) when the plants grew in a soil high in organic matter after a long-term forest-derived fallow than in a soil low in soil organic matter following a long-term savanna-derived fallow (21 t ha^−1^ for *D. alata* and 3.7 t ha^−1^ for *D. rotundata*). Similarly, Kassi et al. (2017) report a positive relationship between soil organic carbon stocks and tuber yields of *D. rotundata* with maximum yields obtained after forest and *Chromoleana odorata* fallows.

Aspects to be considered to understand the importance of soil fertility for yam are: the nutrient uptake in tubers and in the plant at maximum growth, as tuber yield is correlated to the maximum leaf area index (Diby et al., 2011c), the critical nutrient concentration in leaves under which a deficiency will appear and the amount of nutrients that can be released from the soil and taken up by the crop. Many publications report nutrient contents in tuber for N, P, K, Ca, and Mg (Table 1), but very few on micronutrients (Frossard et al., 2000). The results presented in Table 1 allow for calculation of the amount of nutrients exported from the field at tuber harvest. Fewer publications analyzed nutrient uptake and distribution during the entire plant growth (Irizarry and Rivera, 1985; Irizarry et al., 1995; Diby et al., 2011b) and even less were written on the critical nutrient concentration in yam (Shiwachi et al., 2004; O'Sullivan and Jenner, 2006).

**Table 1:** Nutrient concentration, water content, and dry matter tuber yield of *D. alata* and *D. rotundata* grown in various regions.

**Species**	**Cultivar**	**Country**	**Nutrient concentration in yam tuber at harvest, kg t**^**−1**^ **Dry Matter**					**Moisture content in tuber, kg water t^−1^ Fresh Matter**	**Tuber yield, t Dry Matter ha^−1^**	**References**
			**N**	**P**	**K**	**Ca**	**Mg**			
*D. alata*	N/M^a^	Kerala, India	15.9	2.0	20.0	nd^b^	nd^b^	nd^b^	4.6	Kabeerathumma et al., 1991
	Brazo fuerte	Abidjan, Côte d'Ivoire	10.2	1.4	9.6	0.5	0.8	76.7	1.9	Budelmann, 1989
	Okinawa white	Miyako Islands, Japan	12.3	2.2	26.7	3.3	2.5	87.7	0.3	Shiwachi et al., 2015
	Gunung	Puerto Rico	19.0	1.6	19.1	5.0	6.0	85.2	8.7	Irizarry et al., 1995
	N/M	Western Nigeria	14.2	1.9	17.9	0.3	0.9	75.2	6.2	Obigbesan and Agboola, 1978
	TDa95/00010	Centre, Côte d'Ivoire	21.0	1.1	18.5	1.0	1.0	80.0	10.0	Diby et al., 2011b
	**Mean for** ***D. alata***		**15.4**	**1.7**	**18.6**	**2.0**	**2.2**	**81.0**	**5.3**	
	**Mean nutrient export per ton tuber Fresh Matter ha**^−1^		**2.9**	**0.3**	**3.5**	**0.4**	**0.4**			
*D. rotundata*	N/M	Kerala, India	9.1	1.6	10.9	nd^b^	nd^b^	nd^b^	9.7	Kabeerathumma et al., 1991
	Habanero	Puerto Rico	8.1	1.3	9.5	1.0	1.4	65.1	18.0	Irizarry and Rivera, 1985
	Efuru	Western Nigeria	12.8	1.5	14.5	0.3	0.9	67.2	10.4	Obigbesan and Agboola, 1978
	Aro	Western Nigeria	11.5	1.5	12.7	0.3	0.9	65.6	9.7	Obigbesan and Agboola, 1978
	Obiaoturugo	Benin City, Nigeria	2.2	0.2	1.2	nd^b^	nd^b^	63.8	5.5	Law-Ogbomo and Remison, 2009
	TDr95/18544	Ibadan, Nigeria	3.2	1.7	5.9	0.7	1.2	64.0	3.4	Kikuno et al., 2015
	**Mean for** ***D. rotundata***		**7.8**	**1.3**	**9.1**	**0.6**	**1.1**	**65.1**	**9.4**	
	**Mean nutrient export per ton tuber Fresh Matter ha**^−1^		**2.7**	**0.5**	**3.2**	**0.2**	**0.4**			

*N/M^a^, not mentioned; nd^b^ data not provided*.

The amount of soil nutrients taken up by a crop can be derived from trials where nutrient additions are varied. Many trials have been conducted on yam (Carsky et al., 2010; Susan John et al., 2016). Some show positive impacts of N, P, and K inputs on tuber yields, while other do not show any impact of nutrient additions. The results of many trials are however difficult to interpret since numerous factors, often not reported in publications, impact tuber yields. These are weather conditions, cultivar, yam seed quality, seed weight, planting density, planting date, weeds, diseases and pests, and plot history (Rodriguez-Montero et al., 2001; Cornet et al., 2014, 2016). For instance, the heterogeneous germination of yam due to variable yam seed quality leads to a large yield variability that can blur any treatment effect (Cornet et al., 2014). Melteras et al. (2008) observed radial of roots of *D. esculenta* that were longer than 3 m and suggested that this would lead to wrong interpretation of fertilizer trial results as an unfertilized plant would be able to take up nutrients from a fertilized plot. On the contrary, no N transfer was observed from fertilized to unfertilized plots in a study conducted with ^15^N labeled fertilizer on *D. alata* with 1 m space between plots (Hgaza et al., 2012). Except in the work done by Kassi et al. (2017), no relation linking soil properties and yam tuber yield has been published. A prerequisite to understanding the relationships between soil properties and yam yield will be to install field trials on different soils with different rates of nutrient addition using identical crop management techniques together with sufficient information on weather.

Hgaza et al. (2012) observed a positive effect of NPK input on *D. alata* yield in a low fertility savanna. Since the added N was labeled with ^15^N, these authors could show that the fertilizer input had triggered an increased uptake of N derived from the soil by the crop. As this input had not changed root growth (Hgaza et al., 2011), the authors concluded that the NPK addition had increased the rate of soil organic matter mineralization. This phenomenon needs further investigation as it can have negative consequences on these soils, which have very low organic matter contents. Whether such an effect would also occur following organic fertilizer inputs should be assessed. In the same study, Hgaza et al. (2012) observed a maximum N fertilizer recovery of below 30% in the tuber. This limited recovery was explained by the low planting density (one plant m^−2^), which is typical for West Africa and by the coarse and superficial root system of *D. alata* (Hgaza et al., 2011). This low recovery rate suggests high rates of nutrient losses which need to be quantified. Mineral fertilizer inputs have also been reported to increase tuber weight loss and rotting during storage and to negatively affect the organoleptic properties of tubers (Vernier et al., 2000; Baimey et al., 2006). Since fertilizers use will become unavoidable to increase yam productivity, the effects of fertilizer on tuber quality will need to be studied.

Guidelines for yam fertilization are shown in Table 2. These call for some remarks. No distinction is made between yam species except for Sri Lanka. Only Nigeria makes a difference between soil fertility classes, but these classes are not defined with respect to yam. Three of the guidelines recommend also manure and lime applications. No official recommendations were found for West African countries except Nigeria. The guidelines shown in Table 2 seem to cover the N and K needs for a tuber yield of 30 t ha^−1^ while showing a massive P over-fertilization.

**Table 2:** Fertilization guidelines on yam (*Dioscorea* spp.) from different regions.

**Country**		**Target species**	**Target yield**	**Water-soluble fertilizer**				**Lime**	**Manure**	**Timing of NPK addition**	**Other**
				**N**	**P**	**K**	**Mg**				
			**t FM ha**^−1^	**kg ha**^−1^ **year**^−1^				**t ha**^−1^ **year**^−1^			
India	Kerala^a^			80	60	80					
	Tamil Nadu^b^	*D. alata* and	20–25 t ha^−1^	40	60	120			25	Before planting	
		*D. esculenta*									At 30 days after planting: 4 kg ha^−1^ *Azospirillum*
				50		120				90 days after planting	
Nigeria^c^	Low fertility class soil	*D. alata* and	Between 5 and 50 t ha^−1^	90	54	75	6	0.5		56 days after planting	Apply in each case NPK in ring 15 cm from base of
	Medium fertility class soil	*D. esculenta*		45	25	40	6	If soil acidic		56 days after planting	the vine, 3-5 cm deep;
	High fertility class soil			20	0	0	6			56 days after planting	Apply Mg as basal fertilization
Sri Lanka^d^		*D. alata*		30	30	29		1–2	10	Before planting	Incorporate basal NPK into soil before planting
				30		29		If soil acidic		60 days after planting	Subsequent applications to be done in a circle
				30		29				90 days after planting	around the plant and incorporated into soil
French	Martinique	*D. alata*,	Between 15 and 30 t ha^−1^	41	45	113		1–3	20	Before planting	
West		*D. cayenensis*,		69				If soil acidic		45 days after planting	
Indies^e^		and *D. trifida*		20		58				90 days after planting	

a http://www.keralaagriculture.gov.in/htmle/crops/fertiliserrec.htm;

b http://agritech.tnau.ac.in/horticulture/horti_vegetables_Dioscorea.html;

c Chude et al. (2011);

d Department of Agriculture (2007);

e *http://www.martinique.chambagri.fr/fileadmin/documents/CDA_martinique/internet/listes_frontend/SDQ/Fiches_techniques/FIT-Igname14.pdf*.

Water-soluble mineral fertilizers are not often used on yam in West Africa. Alternative to sustain plant nutrition are the use of less demanding yam cultivars, to make a better use of microorganisms fostering plant nutrition, to intercrop yam with legumes, to add organic mulch, or to recycle wastes as sources of nutrients. Current research is identifying cultivars of *D. alata* able to produce large tuber yields when planted in acid, alkaline or saline soils (Perlas et al., 2010; Anyanwu and Ildefonso, 2015; Shiwachi et al., 2015; Takada et al., 2017). Rezaei et al. (2017) recently suggested that the ability of *D. esculenta* to grow under low N conditions would be related to the presence of N-fixing endophytic bacteria able to sustain the N nutrition of the plant. Mycorrhizal fungi can colonize yam roots, and management affects mycorrhizal communities in yam-based systems (Tchabi et al., 2008, 2009; Dare et al., 2013, 2014). However, we still lack knowledge on the impact of mycorrhizae in the field and on how to manage them to improve yam yield. Intercropping yam with herbaceous legumes in the presence of fertilizer increases tuber yield and nutrient recycling rate (Maliki et al., 2012b). However, whether the recycled nutrients are taken up by following crops is not known. Intercropping yam with *Gliricidia sepium* is promising as it can be used as a stake for yam vines while fixing N from the atmosphere (Budelmann, 1990a,b; O'Sullivan et al., 2008). The addition of *G. sepium, Tithonia diversifolia*, or *C. odorota* mulches can also improve yam yield by providing nutrients, limiting weed invasion and decreasing soil temperature (Budelmann, 1989; Agbede et al., 2014). Except from one work showing an increase of *D. rotundata* tuber yield following urine input (Comoé et al., 2009), no other publication was found on the effect of waste recycling on yam. Finally, Agbede (2006) and Agbede and Adekiya (2013) show that mounding or ridging lead to higher tuber yields than no-till and that soil apparent density is negatively correlated to tuber yield.

Beside the approaches already mentioned, farmers have developed strategies to cope with soil fertility depletion. In Benin, these include the selection and cultivation of less demanding yam cultivars, the introduction of yam in rotations to benefit from the residual effect of fertilizers added to previous crops, and the cultivation of yams in sites where water, organic matter, and nutrients accumulate such as, lowlands and old cattle corrals (Floquet et al., 2012). Another example is found in the province of Passoré (Burkina Faso) where yam is grown under semi-arid conditions (700 mm year^−1^) on hydromorphic soils in rotation with other staple crops and with organic and mineral fertilizers (Dumont et al., 2005; Tiama et al., 2016). The impacts on yam yield formation, nutrient dynamics, and use efficiency of these adaptations have not yet been studied.

The ISFM framework has been proposed to increase yields in smallholders' tropical settings. ISFM is based on the combined use of organic and mineral nutrient sources in conjunction with appropriate crop varieties and adaptations to the local context (Vanlauwe et al., 2010, 2015). ISFM is implicitly mentioned in three of the fertilization guidelines (Table 2) and is already practiced in regions of Benin and Burkina Faso as discussed above. ISFM on yam starts to be studied (Ennin et al., 2013; Lawal et al., 2013) but it could be used on a much broader scale. The following section is dedicated to barriers that could limit the adoption of ISFM in yam-based systems of West Africa.

## Barriers that might limit the adoption of ISFM in yam-based systems of West Africa

There is little information on the acceptance of soil management practices for yam (Maliki et al., 2012a) and more generally on the adoption of new technologies in yam (Dao et al., 2003; Soro et al., 2010). Overall, the adoption of new technologies in yam seems limited. For instance, the minisett technology that uses small and healthy tuber parts, and that was developed decades ago (Aighewi et al., 2014), has not been widely adopted (Okoro and Ajieh, 2015). Similarly, high yielding yam varieties tolerant to diseases and growing without staking have not been widely adopted (Alene et al., 2015). Notable exceptions have been the adoption in Côte d'Ivoire of the *D. alata* varieties Florido and C18, which are easy to grow while showing good resistance to diseases (Doumbia et al., 2004, 2014). Moreover, C18 is well appreciated for cooking “foutou,” a yam-based dish (Doumbia et al., 2014), which is a driver for technology adoption in West Africa, as food quality is very important to producers and consumers (Adesina and Baidu-Forson, 1995).

Low adoption rates of soil improving options in yam-based systems have been linked to the fact that researchers neither paid sufficient attention to the multitude of problems farmers really face, nor built on the diversity of problem-solving practices developed by farmers in their diverse biophysical and socio-economic contexts (Nederlof and Dangbégnon, 2007; Floquet et al., 2012). The adoption of ISFM in yam-based systems of West Africa might indeed be challenging for the following reasons. Will yam producers having access to old woody fallows, even though such fallows are becoming scant and remote from villages, find ISFM more efficient in terms of returns to labor? Will it be possible for yam farmers having access to limited land to mobilize sufficient organic resources such as, *G. sepium* for ISFM at reasonable opportunity costs? Will yam farmers, who do not own their land, be motivated to invest in improving its fertility?

Altogether, there is a potential for ISFM in yam-based systems but this needs to be linked to farmers' options and preferences and to the demand expressed by the different actors along the value-chain. Most of the internal (labor, organic matter from planted fallow, or mixed agroforestry component) and external (mineral fertilizers, herbicides, improved planting materials) resources needed to implement ISFM may require investments from the individual farmer or the community which could limit the return on investment and thus the adoption of ISFM practices. Overall, finding out the right mix of ISFM measures will require a high level of collaboration between actors to define a joint intervention strategy and activities to generate scalable outputs built on farmers' experiences and perceptions and suited to the diversity of local contexts. We suggest that IPs could be a mean to define jointly such strategies for reasons given thereafter.

## Innovation platforms as a tool to develop collaboration between actors for designing innovations in yam-based systems

“Innovation platforms (IPs) are a way of organizing multi-stakeholder interactions, marshaling ideas, people and resources to address challenges and opportunities embedded in complex settings” (Davies et al., in press). IPs are often organized around a farm product, such as, yam (Bonfoh et al., 2016), and include relevant stakeholders connecting households and community operational settings with state policies and institutions. Experiences with IPs reveal that they both affect market connections and technological knowledge within the product value chain (Adekunle et al., 2012). Jiggins et al. (2016) summarizing the results from a range of well documented IPs in West Africa pinpoint the importance of building trust for shared action and of shared learning in experimental processes of change. Hounkonnou et al. (in press) conclude from their experiences with nine IPs that the design can help leverage institutional constraints and create favorable niches of change. Whether such niches can trigger changes in the technological and institutional regimes that are needed to develop a prosperous yam economy making a sustainable use of natural resources must still be proven. There are few published reports on how the work of IPs can be used to foster sustainable soil fertility management (Tittonell et al., 2012). But, no publication was found on how IPs could foster sustainable soil fertility management in tropical root and tuber crops such as yam.

## Recommendations for future research

This review showed that improving soil fertility in yam-based systems in West Africa faces three challenges that need to be addressed simultaneously if research is to deliver soil management innovations that are sustainable, feasible, and acceptable. These challenges are: (i) improving our understanding of the relations between soil properties, management, and tuber yield, (ii) analyzing the social and economic impacts of these innovations, and (iii) assessing their acceptance and implementation by stakeholders.

We recommend future research to take the following steps to address these challenges. Research must be conducted in a transdisciplinary manner involving the relevant actors from the practice (Baveye et al., 2014), from the problem definition, to the co-design of soil management innovations, the evaluation of research results, their communication, and their implementation. This could be done by fostering IPs including producers, actors involved in the yam-value chain as well as authorities, the media, microcredit organizations, and agricultural extension agencies as all of them will influence the decision of farmers to implement innovative soil management. The research should be conducted by interdisciplinary teams including experts in natural and social sciences. The co-designed soil management innovations should be tested following the mother/baby trials scheme (Snapp, 2002). This work should be done in sites showing a large diversity in terms of their biophysical and socio-economic characteristics to derive generic rules on soil/plant relationships in yam as affected by soil management and on how to develop and adjust the innovation supply to specific contexts. Finally, research will have to trigger collaboration with so-called organizations of change such as, national agricultural extension agencies to out and upscale the approach and options developed by research.

## Author contributions

EF led the review. All co-authors except RS made substantial contributions to the conception, development and revision of this review during the various workshops of the YAMSYS project. RS made substantial contributions for the acquisition of data for the revised version and contributed critically to the revised version. All co-authors approved the final version and agreed to be accountable for all aspects of this work.

### Conflict of interest statement

The authors declare that the research was conducted in the absence of any commercial or financial relationships that could be construed as a potential conflict of interest.
